# Unsupervised Black-Box Model Domain Adaptation for Brain Tumor Segmentation

**DOI:** 10.3389/fnins.2022.837646

**Published:** 2022-06-02

**Authors:** Xiaofeng Liu, Chaehwa Yoo, Fangxu Xing, C.-C. Jay Kuo, Georges El Fakhri, Je-Won Kang, Jonghye Woo

**Affiliations:** ^1^Gordon Center for Medical Imaging, Massachusetts General Hospital and Harvard Medical School, Boston, MA, United States; ^2^Department of Electronic and Electrical Engineering and Graduate Program in Smart Factory, Ewha Womans University, Seoul, South Korea; ^3^Department of Electrical and Computer Engineering, University of Southern California, Los Angeles, CA, United States

**Keywords:** unsupervised domain adaptation, black-box model, segmentation, brain tumor, MR image, knowledge distillation

## Abstract

Unsupervised domain adaptation (UDA) is an emerging technique that enables the transfer of domain knowledge learned from a labeled source domain to unlabeled target domains, providing a way of coping with the difficulty of labeling in new domains. The majority of prior work has relied on both source and target domain data for adaptation. However, because of privacy concerns about potential leaks in sensitive information contained in patient data, it is often challenging to share the data and labels in the source domain and trained model parameters in cross-center collaborations. To address this issue, we propose a practical framework for UDA with a black-box segmentation model trained in the source domain only, without relying on source data or a white-box source model in which the network parameters are accessible. In particular, we propose a knowledge distillation scheme to gradually learn target-specific representations. Additionally, we regularize the confidence of the labels in the target domain via unsupervised entropy minimization, leading to performance gain over UDA without entropy minimization. We extensively validated our framework on a few datasets and deep learning backbones, demonstrating the potential for our framework to be applied in challenging yet realistic clinical settings.

## 1. Introduction

Semantic segmentation provides the pixel-wise annotation of lesions or anatomical structures and has been an important prerequisite for early diagnosis and treatment planning (Liu et al., 2020a,b; He et al., 2022). Because of the high cost of manual delineations, there is a large demand for automatic segmentation tools for clinical practice. For the past several years, with the development of data-driven deep learning, the performance of segmentation tasks has been substantially improved (Liu et al., 2020c, 2021h). For example, U-Net and its follow-up backbones achieved outstanding performance compared with their predecessors, in many natural and medical image analysis tasks, including the brain tumor localization and segmentation from magnetic resonance (MR) images (MRI) (Liu et al., 2020d; He et al., 2022).

The performance of a pre-trained deep learning model, however, can be substantially degraded, when its training distribution (i.e., source domain) differs from a testing distribution (i.e., target domain). This is because the majority of deep learning architectures assume that the source and target data distributions are independent and identically distributed (*i*.*i*.*d*.) and thus invariant across domains. This assumption, however, is deemed unrealistic in many clinical settings. For example, tumors with different grades are likely to exhibit different data distributions, due to varying degrees of tumor severity and growth patterns (Liu et al., 2021j). In addition, in cross-center collaborations, data acquired even with the same vendor and with the same acquisition protocol can be substantially different from one another. Furthermore, under many multimodal MR image segmentation scenarios, cross-modality domain shifts, e.g., T2-weighted to T1-weighted MRI, can arise, leading to large performance degradation.

To accommodate the difference in distributions between training and testing data, a possible solution is to fine-tune developed models with supervised training, which requires pixel-wise ground truth labeling in the target domain. Since it is costly to annotate high-quality labeled data in new target domains, unsupervised domain adaptation (UDA) has been developed (Liu et al., 2021e) to adapt the model trained in a labeled source domain to different and unlabeled target domains. In the conventional UDA, segmentation models have been trained using both source and target data, but only the source data are labeled at the adaptation stage. Promising results have been reported by means of co-training models with source domain data, primarily by enforcing the similar feature distribution of source and target domains with maximum mean discrepancy minimization (Long et al., 2015), adversarial training (Liu et al., 2021a), and self-training (Zou et al., 2019).

Although UDA offers a promising solution to the problem of domain shift, because of privacy concerns about sensitive patient data being leaked, it is often challenging to access data and their labels in the source domain and trained model parameters in cross-center collaborations (Liu et al., 2021k). Cross-center data sharing usually requires sophisticated anonymous processing and ethics approvals, which can hinder fast deployment. In addition, large-scale and well-labeled medical datasets can be a valuable core competence for both research and commercial institutes. To address this issue, Liu et al. (2021k) have proposed a source-free or source-relaxed UDA approach (i.e., white-box domain adaptation) for segmentation. In that work, an off-the-shelf segmentation model was adapted to a target domain via a pre-trained model in a source domain, by transferring its batch normalization statistics. Recently, a deep inversion technique (Yin et al., 2020) has shown that original training data can be recovered from knowledge used during white-box domain adaptation, which may leak confidential information and raise privacy concerns over patient data (Zhang et al., 2021). In addition, source-free UDA usually relies on the same network structure as in the trained source domain, which is not flexible to update state-of-the-art or lightweight backbones to achieve better performance or implementation on memory-limited mobile devices.

This study aims to overcome these limitations by developing a black-box domain adaptation approach, in which we opt to restrict the use of knowledge from a source segmentation model, and do not rely on the network parameters. As a result, we provide stricter protection of medical data privacy. In addition, public release of large-scaled trained and packaged models can be easily applied to task-specific adaptation, such as segmentation and classification. To the best of our knowledge, this is the first attempt at achieving UDA for deep segmentation networks using black-box domain adaptation. Our prior work showed an initial network design and concept (Liu et al., 2022). Building upon that work, the present study describes refined network architectures and provides extensive validations on a few different datasets and network backbones. The black-box setting provides a more effective way to protect privacy, compared with white-box domain adaptation approaches (Liu et al., 2021k) or conventional UDA approaches (Zou et al., 2019). To our knowledge, no prior work has yet been reported on recovering data from a “black-box” model. Recently, Zhang et al. (2021) proposed to use black-box UDA for classification, with class-wise noise rate estimation and category-wise sampling. That work presented iterative learning with noisy labels, in which the black-box predictions were considered noisy labels. However, that work cannot be directly applied to the segmentation task to perform pixel-wise classification. Additionally, a few attempts have been made to carry out black-box domain adaptation (Liu et al., 2022), although they could be used in more challenging yet realistic clinical scenarios.

## 2. Related Work

### 2.1. Semantic Segmentation

The fully convolutional network (FCN) (Long et al., 2015) was a pioneering work of deep semantic segmentation. Then, the Pyramid Scene Parsing Network (PSPNet) (Zhao et al., 2017) was proposed to exploit the spatial feature at different scales of FCN. Recently, U-Net (Ronneberger et al., 2015) has been widely used as the backbone of many segmentation networks, which have skip connections between the encoder and decoder to adaptively learn the correlations at different resolution scales. Other than the conventional convolutional layers used in vanilla U-Net, more advanced versions of U-Net, including ResNet (He et al., 2016) and MobileNet (Howard et al., 2017) have also been proposed to further boost performance or efficiency. Our black-box UDA framework is agnostic to any segmentation network, where the network used in source and target domains can be different to fit into specific requirements in implementation.

### 2.2. Unsupervised Domain Adaptation

Unsupervised domain adaptation (He et al., 2020a,b; Liu et al., 2021c,e) has been an important technology to alleviate the problem of domain shift and costly labeling in a new domain. Conventional approaches have utilized both source and target domain data for training (Liu et al., 2021a,d,g,i,j). Recently, source-free UDA (Bateson et al., 2020; Liang et al., 2020; Wang et al., 2020) has been proposed, which uses a pre-trained model rather than co-training the network with source and target domain data. We note that domain generalization (Liu et al., 2021b), a closely related but different task, assumes that there are no target domain data in its training. A recent work (Liu et al., 2021f) explored shared or domain-specific batch-normalization statistics to achieve domain alignment.

### 2.3. Model Transfer

Early works (Joachims et al., 1999; Duan et al., 2009) for adapting a model with parameters attempted to transfer a trained source classifier with a subset of labeled samples, which is only applicable for semi-supervised adaptation tasks. Kuzborskij and Orabona (2013) proposed a detailed theoretical analysis of hypothesis transfer learning for linear regression, which is the basis for subsequent UDA solutions that do not rely on source data at the adaptation stage (Chidlovskii et al., 2016). In the deep learning era, Liang et al. (2020) proposed to fix the last few layers by turning the feature extraction parts into information maximization and pseudo-label-based self-training. Recently, Li et al. (2020) proposed using conditional generative adversarial networks (GAN) to generate images at the adaptation stage. Similarly, Kundu et al. (2020) utilized GAN to explore conditional entropy. However, all of the above methods require knowledge of the network parameters, which thereby can be regarded as white-box source-free UDA.

### 2.4. Knowledge Distillation

Knowledge distillation is proposed to transfer knowledge learned by a teacher model to a student model. Typically, the teacher model has larger backbones with more parameters, while the student one is typically a more compact model. Therefore, it is possible to efficiently compact a model with little sacrifice of performance. The conventional solution used a distillation loss function to enforce the consistency between the outputs of teacher and student models with the same input sample (Hinton et al., 2015). Essentially, the knowledge distillation is an adaptive label smoothing regularization (Szegedy et al., 2016). Kim et al. (2020) showed that the previous prediction can teach the network with a self-knowledge distillation scheme, which can be potentially used for semi-supervised learning. A recent work (Samuli and Timo, 2017) assembled the prediction along with the training as a teacher model prediction. Rather than using the average teacher model predictions, Tarvainen and Valpola (2017) used the averaged previous model parameters as a teacher model.

## 3. Methodology

Image segmentation partitions medical images into coherent regions for different lesions or anatomical structures, and is essential for many computer-aided diagnosis systems. A typical solution would be to formulate the segmentation task as a pixel-wise classification. *f*_*s*_ takes an encoder and decoder structure to map an input image, e.g., an MRI slice in the BraTS database $x_{s}\in {\mathbb{R}}^{128\times 128}$, to its corresponding segmentation map $y_{s}\in {\mathbb{R}}^{128\times 128\times C}$, where *C* is the number of classes.

Considering the potential distribution shift between two domains, we assume that there are a source domain *p*_*s*_(*x, y*) and a target domain *p*_*t*_(*x, y*), where *x* indicates the to be segmented image and *y* is its corresponding label of the segmentation map. In the setting of black-box UDA segmentation, we have a segmentation network *f*_*s*_ trained with a labeled source domain set $D_{S}=\{x_{s},y_{s}\}$ drawn *i*.*i*.*d*. from *p*_*s*_(*x, y*), where *f*_*s*_ is fixed and accessed only through a nontransparent API during the adaptation stage. At the adaptation stage, we only have access to a black-box *f*_*s*_ and an unlabeled target domain set $D_{T}=\{x_{t}\}$ drawn *i*.*i*.*d*. from the marginal distribution *p*_*t*_(*x*), to train a target domain network *f*_*t*_ to achieve a good segmentation performance in the target domain. It is noteworthy that the backbones of *f*_*s*_ and *f*_*t*_ do not need to be the same. The network structure details may also not be available at the adaptation stage.

**Figure 1:**
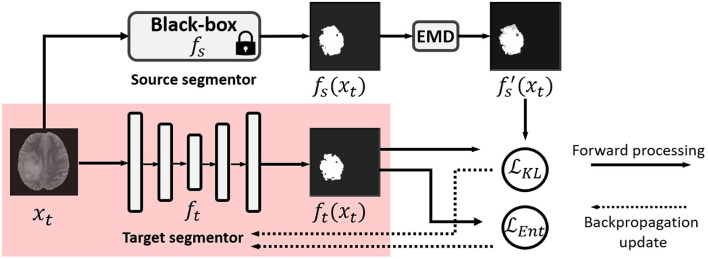
Illustration of our black-box UDA framework using knowledge distillation with exponential mixup decay (EMD) pseudo label and unsupervised entropy minimization. Only the red shaded parts are used in testing in the target domain. Note that *f*_*s*_ and *f*_*t*_ can have different backbones.

In this work, we propose a practical solution to black-box UDA for segmentation with a noise-aware knowledge distillation scheme using pseudo labels with exponential mixup decay (EMD). The framework is shown in Figure 1.

### 3.1. Supervised Source Domain Training

A good source model is a basis for the target domain adaptation performance. The UDA is motivated by the following theorem (Kouw, 2018):

**Theorem 1** For a hypothesis *h*


(1) $$\begin{array}{l} L_{t}\left(h\right)\leq L_{s}\left(h\right)+d\left[p_{s}\left(x\right),p_{t}\left(x\right)\right]+\epsilon , \end{array}$$

where $L_{s}\left(h\right)$ and $L_{t}\left(h\right)$ denote the expected loss with hypothesis *h* in the source and target domains, respectively, and *d*[·] measures the divergence of the marginal distributions of *x* between two domains (Salimans et al., 2016). We note that the last term ϵ = min[*E*_*x*~_*p*__*s*__|*p*_*s*_(*y*|*x*) − *p*_*t*_(*y*|*x*)|, *E*_*x*~_*p*__*t*__|*p*_*s*_(*y*|*x*) − *p*_*t*_(*y*|*x*)|] is usually a small value and does not affect the performance. A small $L_{s}\left(h\right)$ is essential to achieve a low $L_{t}\left(h\right)$, i.e., high accuracy in the target domain.

For the supervision of training, a cross-entropy (CE) loss is usually used for optimization. Specifically, the pixel-wise CE loss can be formulated as:


(2) $$\begin{array}{l} L_{CE}=\frac{1}{H_{0}\times W_{0}}\overset{H_{0}\times W_{0}}{\underset{n=1}{\sum }}\left\{-\frac{1}{C}\overset{C}{\underset{i=1}{\sum }}{y_{s}}_{n}^{i}\log f_{s}{\left(x_{s}\right)}_{n}^{i}\right\}, \end{array}$$

where *H*_0_ and *W*_0_ are the height and width of an image, *n* indexes the pixel, and *i* indexes class labels. We note that other loss functions can also be used, e.g., dice loss, IoU loss, and boundary loss (Jadon, 2020). The supervised source domain training is independent of the adaptation stage in our black-box UDA setting. We note that we do not re-train or fine-tune the fixed black-box source segmentation model.

### 3.2. Knowledge Distillation With Exponential Mixup Decay

Following knowledge distillation (Yin et al., 2020), the well-trained source model can act as a teacher to provide its pixel-wise softmax histogram prediction of each image. The target domain model *f*_*t*_ is trained to imitate the source model *f*_*s*_. The consistency of their predictions can be enforced with the Kullback-Leibler (KL) divergence between their pixel-wise softmax histogram distributions. In the conventional knowledge distillation, we assume that there is no domain shift, and the predictions of the teacher model can be reliable and simply be used as ground truth. However, due to the domain shift, the prediction of *f*_*s*_ in the target domain can be noisy. Simply using it as ground truth cannot outperform the source models, which is not expected in the UDA setting.

Accordingly, we resort to a self-training scheme (Liu et al., 2021j) to construct the pseudo label for target domain training. Considering that the source model predictions can be a relatively reliable supervision signal, compared with unsupervised objectives in the initial epochs, we propose adjusting the contribution of the supervision signals as the training progresses. Specifically, to achieve the gradual translation to the target domain, we mix up the source and target domain predictions, i.e., *f*_*s*_(*x*_*t*_) and *f*_*t*_(*x*_*t*_), and adjust their ratio for the pseudo label $y_{t}^{\prime }$ with EMD:


(3) $$\begin{array}{l} {y_{t}^{\prime }}_{n}=\lambda f_{s}{\left(x_{t}\right)}_{n}+\left(1-\lambda \right)f_{t}{\left(x_{t}\right)}_{n},\\ \lambda =\lambda^{0}\text{exp}\left(-I\right), \end{array}$$

where *n* indexes the pixel, and *f*_*s*_(_*x*_*t*_)*n*_ and *f*_*t*_(_*x*_*t*_)*n*_ are the histogram distributions of the softmax output of the *n*-th pixel of the predictions *f*_*s*_(*x*_*t*_) and *f*_*t*_(*x*_*t*_), respectively. λ is the target adaptation momentum parameter with the exponential decay with respect to iteration *I*. λ^0^ is the initial weight of *f*_*s*_(*x*_*t*_), which is empirically set to 1. Therefore, along with the increase in iteration *I*, we have smaller λ, which adjusts the contribution of the source model prediction to be large at the start of the training and to be smaller at the later training epochs. The loss knowledge distillation with the EMD pseudo label can be formulated as:


(4) $$\begin{array}{l} L_{KL}=\frac{1}{H_{0}\times W_{0}}\overset{H_{0}\times W_{0}}{\underset{n=1}{\sum }}D_{KL}\left(f_{t}{\left(x_{t}\right)}_{n}||{y_{t}^{\prime }}_{n}\right), \end{array}$$

where *H*_0_ and *W*_0_ are the height and width of the image. We note that the KL divergence is a measure of how a probability distribution, e.g., the histogram distribution of *f*_*t*_(_*x*_*t*_)*n*_, is different from a reference probability distribution, e.g., the histogram distribution of ${y_{t}^{\prime }}_{n}$. Minimizing the KL divergence explicitly enforces the similarity of the two distributions of the predictions. Therefore, the weight of λ can be smoothly decreased along with the training, and *f*_*t*_ gradually represents the target data.

### 3.3. Self-Entropy Minimization

In addition to the supervision signal provided by the source domain black-box model, we opt to explore unsupervised learning protocols for unlabeled target domain data. Unsupervised learning has a long history, and there are a number of possible solutions for segmentation. Among them, entropy minimization (Grandvalet and Bengio, 2005) can be an efficient unsupervised training scheme for deep learning-based segmentation. Since it does not need a modification to the networks, it can be a simple add-on loss function on top of our framework. For implementation, the entropy for pixel segmentation can be formulated as the averaged entropy of the pixel-wise softmax prediction, given by


(5) $$\begin{array}{l} L_{Ent}=\frac{1}{H_{0}\times W_{0}}\overset{H_{0}\times W_{0}}{\underset{n=1}{\sum }}\left\{-f_{t}{\left(x_{t}\right)}_{n}\text{log}f_{t}{\left(x_{t}\right)}_{n}\right\}. \end{array}$$

Minimizing $L_{Ent}$ leads to the output *f*_*t*_(_*x*_*t*_)*n*_ close to a one-hot distribution, i.e., confident prediction. The unsupervised learning is combined collaboratively with the black-box source model supervision to update the target model.

### 3.4. Overall Training Protocol

In summary, our training objective can be formulated as


(6) $$\begin{array}{l} L=L_{KL}+\alpha L_{Ent}, \end{array}$$

where α is used to balance between the knowledge distillation with the EMD pseudo label and the entropy minimization scheme. Since the entropy minimization may lead to a trivial solution in that the prediction of any unlabeled target samples is the same one-hot prediction (Grandvalet and Bengio, 2005), we adopt a simple yet effective solution to stabilize the training, by linearly decreasing the hyper-parameter α from 5 to 0 along with the training.

## 4. Experiments and Results

### 4.1. Dataset and Data Split

**Figure 2:**
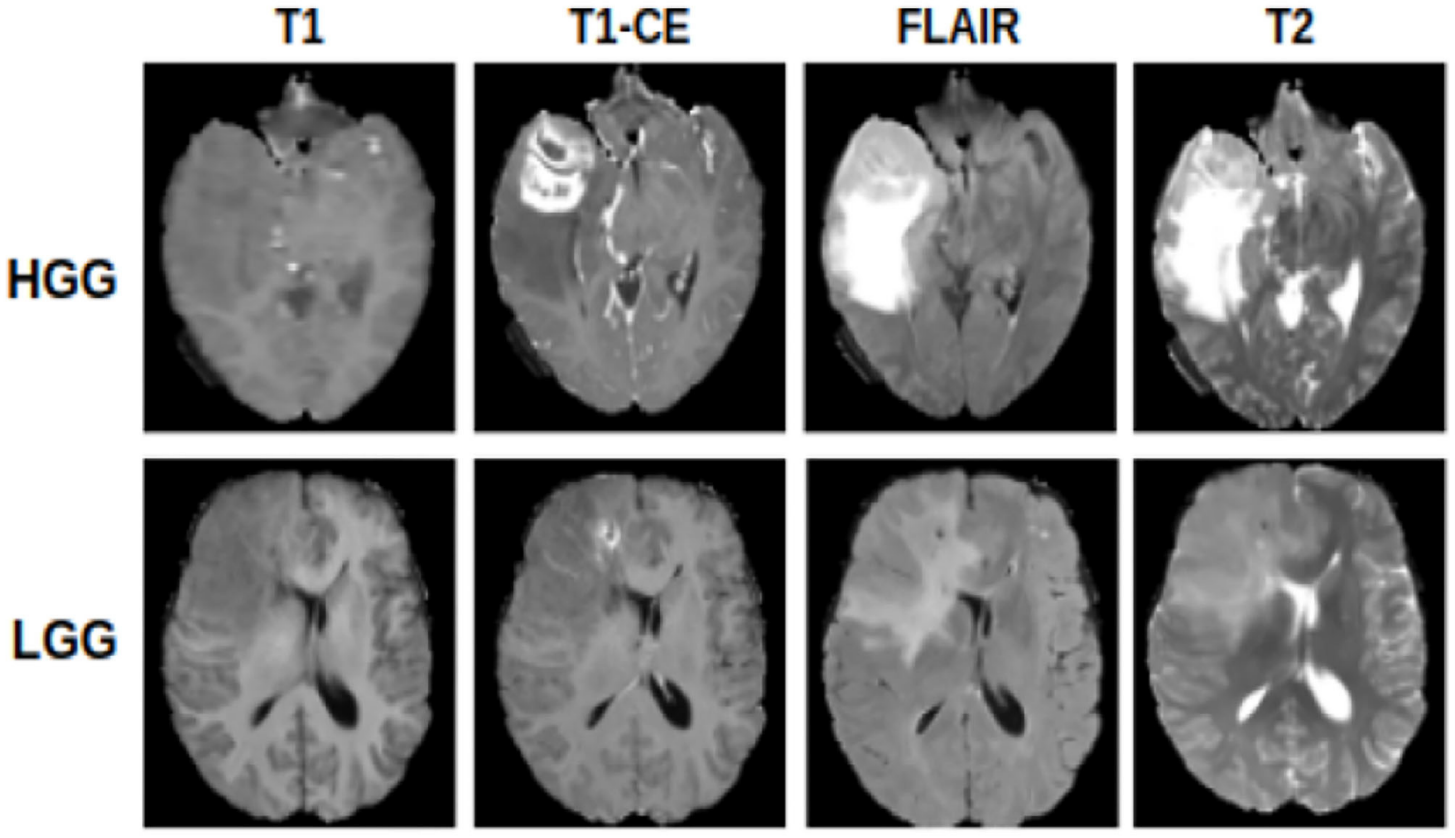
Examples of MRI slices of LGG and HGG samples. Each sample has four MR modalities, i.e., T1-weighted MRI, T1ce MRI, FLAIR MRI, and T2-weighted MRI.

We evaluated our approach on the BraTS2018 database (Menze et al., 2014). In this work, we used a total of 75 patients who have low-grade gliomas (LGG), and a total of 210 patients who have high-grade gliomas (HGG) (Menze et al., 2014) as shown in Figure 2. As a preprocessing step, all of the imaging modalities for each subject were registered with each other, including T1-weighted (T1), T1-contrast enhanced (T1ce), T2-weighted (T2), and T2 Fluid Attenuated Inversion Recovery (FLAIR) MRI. In addition, the voxel-wise labels for the enhancing tumor (EnhT), the peritumoral edema (ED), and the necrotic and non-enhancing tumor core (CoreT) were provided. The whole tumor includes the EnhT, ED, and CoreT. More information about the database can be found in Menze et al. (2014). The source and target domains have the same classes, e.g., CoreT, EnhT, ED, and background.

Following the previous white-box source free UDA (Liu et al., 2021k) and UDA with source data (Shanis et al., 2019), there are two evaluation protocols for UDA, i.e., cross-subtype and cross-modality UDA segmentation. In the cross-subtype setting, we used the HGG subjects as the source domain, and the LGG subjects as the target domain, which have different size and position distributions (Shanis et al., 2019). The slices of the four modalities were concatenated as a 4-channel input with a spatial size of 128×128. The training of adaptation used the LGG training set. We followed the training and testing split as in Liu et al. (2021k). In the cross-modality setting, we used T1- or T2-weighted MRI as the source or target domain, which has a larger domain shift compared with the cross-subtype setting. Each input sample had a single modality slice with the spatial size of 128×128.

### 4.2. Training Protocol and Evaluation Metrics

For the cross-subtype UDA, we experimented on both HGG-to-LGG and LGG-to-HGG tasks. For the HGG-to-LGG task, our training set had a total of 210 labeled HGG subjects as the source domain and a total of 55 unlabeled LGG subjects as the target domain. The remaining 5 and 15 LGG subjects were used as the validation and testing sets, respectively. For the LGG-to-HGG task, our training set had a total of 75 labeled LGG subjects as the source domain and a total of 160 unlabeled HGG subjects as the target domain. The remaining 10 and 40 HGG subjects were used as the validation and testing sets, respectively.

For the cross-modality UDA, we experimented on both T2-to-T1 and T1-to-T2 tasks. For the T2-to-T1 task, our training set had a total of 55 labeled T2 subjects as the source domain and a total of 55 unlabeled T1 subjects as the target domain. The remaining 5 and 15 T1 subjects were used as the validation and testing sets, respectively. For the T1-to-T2 task, our training set had a total of 55 labeled T1 subjects as the source domain and a total of 55 unlabeled T2 subjects as the target domain. The remaining 5 and 15 T2 subjects were used as the validation and testing sets, respectively.

We trained *f*_*s*_ using our prior work (Liu et al., 2021k) and did not have access to its network parameters and source domain data at the adaptation stage. All of the networks used were based on 2D U-Net as in the previous works of UDA using the BraTS18 database (Shanis et al., 2019). Each subject contained a total of 155 MRI slices for each modality. We used U-Net with 15 convolutional layers alongside batch normalization. Of note, for *f*_*s*_, we can use different segmentation backbones as the source domain model. We evaluated two settings that use the same U-Net as *f*_*s*_, and a 15-layer MobileNet-based U-Net. It is of note that MobileNet-based U-Net requires 10× fewer parameters, which is attributed to its separable convolutional operations. It is, therefore, easier for training, requiring much fewer parameters, which has been demonstrated in segmentation tasks on natural images. We used the validation set to tune our parameters. For both source domain only pre-training and adaptation, we used 100 epochs.

The target network at the adaptation stage was trained using Adam as an optimizer with β_1_ = 0.9 and β_2_ = 0.99. The training was performed on four NVIDIA TITAN Xp GPUs with the PyTorch deep learning toolbox (Paszke et al., 2017), which took about 5 h for the cross-subtype task and 4 h for the cross-modality task.

The U-Net with ResNet-15 took about 5 h for the cross-subtype task and 4 h for the cross-modality task. In contrast, the Mobilenet-based U-Net took about 5 h for the cross-subtype task and 4 h for the cross-modality task. For testing, the ResNet and MobileNet based U-Net took about 15 ms and 8 ms for each slice, respectively.

The small size of MobileNet makes it possible to implement MobileNet on some memory-restricted portable devices, e.g., smartphones. We note that the use of MobileNet is to show that we do not need to use and know the same network and the network details, respectively, in the “black-box” case.

For evaluation, we adopted two metrics including Dice similarity coefficient (DSC) and Hausdorff distance (HD) metrics (Zou et al., 2020). The DSC or Sørensen-Dice index, measures the similarity between two sets of data, e.g., pixel set in the image. DSC has been a widely used metric for evaluating image segmentation models. Specifically, it can be formulated as


(7) $$\begin{array}{l} DSC\left(ỹ,y\right)=\frac{2\times |ỹ\cap y|}{\left|ỹ|+|y\right|}. \end{array}$$

The HD between two point sets is defined by the sum of all minimum distances from all points from a point set to another, divided by the number of points in a point set. HD is more sensitive than DSC in terms of the segmentation boundary. In our image segmentation task, the point sets represent the voxels of the ground truth and the segmentation result, respectively, which indicates the maximum HD between the labeled boundary and the predicted boundary.

### 4.3. Evaluation Results

**Figure 3:**
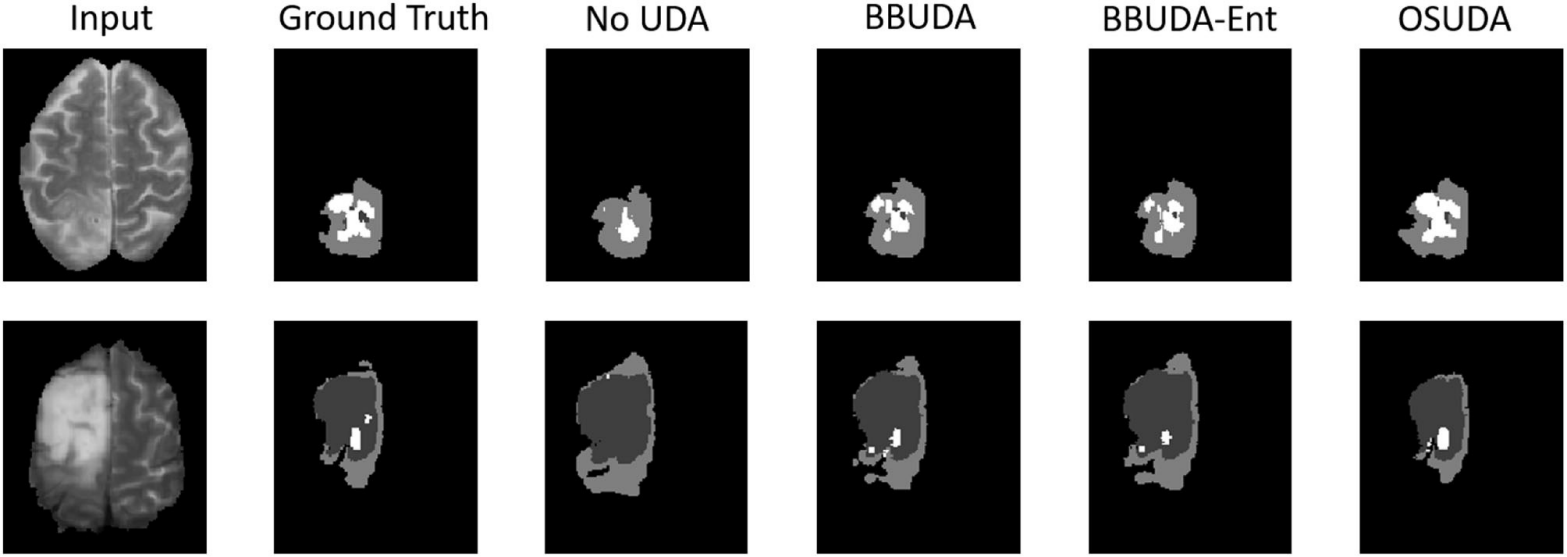
Examples of our segmentation results from an LGG MRI slice with different methods in the HGG to LGG UDA task. In addition, BBUDA-Ent represents an ablation study of the entropy minimization. We use white, dark gray, and gray color to indicate the CoreT, EnhT, and ED, respectively. Of note, OSUDA (Liu et al., 2021k) with the white-box source model for adaptation is considered an “upper bound.”

The segmentation results of different methods are shown in Figure 3. BBUDA and BBUDA-Ent indicate our black-box UDA framework and the ablation study without entropy minimization, respectively. We can see that the predictions of our proposed BBUDA outperform the no adaptation model by a large margin. The better performance of BBUDA over BBUDA-Ent demonstrates the effectiveness of our entropy minimization. In addition, BBUDA+MobileNet indicates using the MobileNet-based U-Net as a segmentor, which has a different structure than the source domain model.

For the cross-subtype UDA task, the quantitative evaluation results of the HGG-to-LGG and LGG-to-HGG tasks are shown in Tables 1, 2, respectively. Our proposed BBUDA achieved the state-of-the-art performance for the black-box source-free UDA segmentation, approaching the performance of the white-box OSUDA (Bateson et al., 2020; Liu et al., 2021k) with the source model parameters, which can be considered an “upper-bound.” We note that the labeling ratio consistency assumption in CRUDA (Bateson et al., 2020) does not hold in this HGG to LGG transfer task, which thus leads to inferior performance. The qualitative evaluation results are shown in Figure 3.

**Table 1:** Quantitative comparisons w.r.t. DSC and HD of HGG to LGG black-box.

**Method**	**Source**	**Dice score [%]** ↑			**Hausdorff distance [mm]** ↓		
	**model**	**WholeT**	**EnhT**	**CoreT**	**WholeT**	**EnhT**	**CoreT**
Source only (Liu et al., 2021k)	no UDA	79.29	30.09	44.11	38.7	46.1	40.2
BBUDA	black-box	82.21	31.33	46.64	28.6	26.4	28.1
BBUDA-Ent	black-box	81.84	31.26	45.75	29.4	27.5	29.0
CRUDA (Bateson et al., 2020)	white-box	79.85	31.05	43.92	31.7	29.5	30.2
OSUDA (Liu et al., 2021k)	white-box	83.62	32.15	46.88	27.2	23.4	26.3
BBUDA+MobileNet	black-box	81.84	31.25	46.16	28.9	26.8	28.3
OSUDA+MobileNet	white-box	82.67	32.09	46.59	28.1	24.2	26.7

*Source only model indicates directly using the source domain trained model in the pre-training step without the subsequent adaptation steps*.

*Of note, OSUDA (Liu et al., 2021k) with the white-box source model for adaptation is considered an “upper bound.”*

**Table 2:** Quantitative comparisons w.r.t. DSC and HD of LGG to HGG black-box.

**Method**	**Source**	**Dice score [%]** ↑			**Hausdorff distance [mm]** ↓		
	**model**	**WholeT**	**EnhT**	**CoreT**	**WholeT**	**EnhT**	**CoreT**
Source only (Liu et al., 2021k)	no UDA	81.45	34.36	40.30	36.7	41.6	37.2
BBUDA	black-box	85.47	39.56	45.18	26.7	33.8	29.6
BBUDA-Ent	black-box	84.92	38.64	44.73	27.1	34.6	31.3
CRUDA (Bateson et al., 2020)	white-box	87.62	40.17	49.65	23.9	22.7	23.9
OSUDA (Liu et al., 2021k)	white-box	89.75	44.21	50.34	22.2	19.3	21.6
BBUDA+MobileNet	black-box	82.14	31.02	46.13	27.2	33.0	28.2
OSUDA+MobileNet	white-box	83.36	31.84	46.62	23.5	21.6	22.8

*Source only model indicates directly using the source domain trained model in the pre-training step without the subsequent adaptation steps*.

*Of note, OSUDA (Liu et al., 2021k) with the white-box source model for adaptation is considered an “upper bound.”*

**Figure 4:**
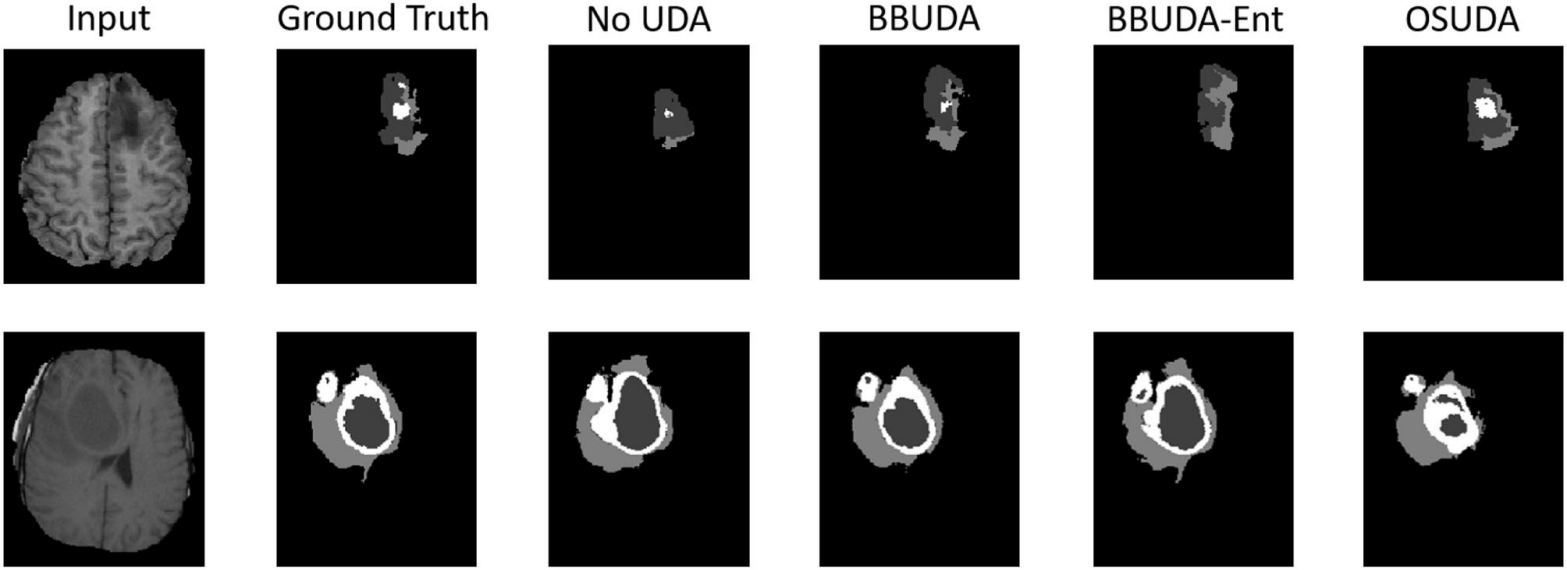
Examples of our segmentation results from T1-weighted MRI with different methods in the T2 to T1 UDA task. In addition, BBUDA-Ent represents an ablation study of the entropy minimization. We use white, dark gray, and gray color to indicate the CoreT, EnhT, and ED, respectively. OIt is of note that OSUDA (Liu et al., 2021k) with the white-box source model for adaptation is considered an “upper bound.”

**Figure 5:**
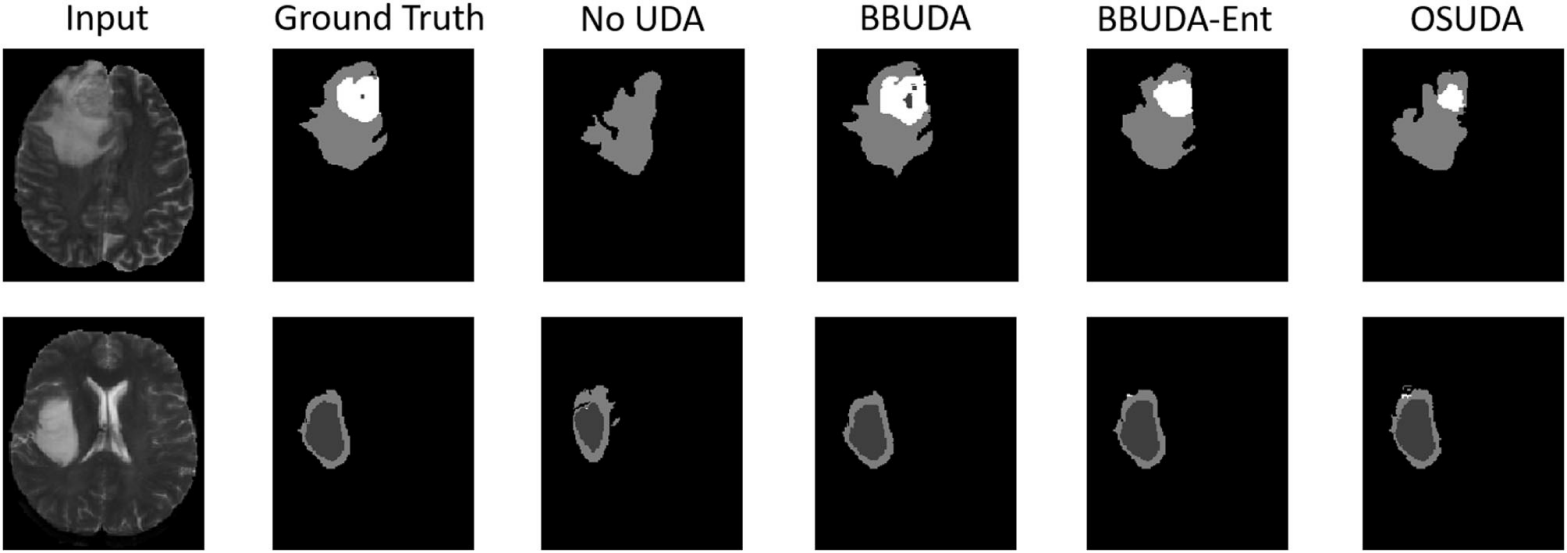
Examples of our segmentation results from T2-weighted MRI with different methods in the T1 to T2 UDA task. In addition, BBUDA-Ent represents an ablation study of the entropy minimization. We use white, dark gray, and gray color to indicate the CoreT, EnhT, and ED, respectively. OSUDA (Liu et al., 2021k) with the white-box source model for adaptation is considered an “upper bound.”

For the cross-modality UDA task, we provide the quantitative evaluation results of T2 to T1 and T1 to T2 in Tables 3, 4, respectively. In addition, the qualitative evaluations are shown in Figures 4, 5. Our proposed BBUDA improved performance in the target domain and outperformed the source model by a large margin. Both the DSC and HD metrics of our framework approached those of the “white-box” model. The sensitivity study of α is provided in Table 5. We found that decreasing the value of α yielded better performance than using a constant value of α.

**Table 3:** Comparison of T2 to T1 black-box UDA.

**Method**	**Source**	**Dice score [%]** ↑			**Hausdorff distance [mm]** ↓		
	**model**	**WholeT**	**EnhT**	**CoreT**	**WholeT**	**EnhT**	**CoreT**
Source only (Liu et al., 2021k)	no UDA	54.50	29.62	23.18	42.7	46.4	44.5
BBUDA	black-box	78.35	36.17	39.28	34.6	38.5	36.8
OSUDA (Liu et al., 2021k)	white-box	79.24	36.43	40.10	32.3	36.7	35.4
BBUDA+MobileNet	black-box	77.62	35.36	38.45	36.4	39.5	37.1
OSUDA+MobileNet	white-box	78.37	36.28	39.84	35.2	39.0	36.3

*The source model is trained with T2-weighted MRI slices, and the testing input is T1-weighted MRI slices. OSUDA (Liu et al., 2021k) with the white-box source model for UDA training is regarded as an “upper bound.”*

**Table 4:** Comparison of T1 to T2 black-box UDA.

**Method**	**Source**	**Dice score [%]** ↑			**Hausdorff distance [mm]** ↓		
	**model**	**WholeT**	**EnhT**	**cCoreT**	**WholeT**	**EnhT**	**CoreT**
Source only (Liu et al., 2021k)	no UDA	52.61	20.44	22.69	45.3	47.8	40.9
BBUDA	black-box	76.26	37.30	39.57	39.5	42.3	33.7
OSUDA (Liu et al., 2021k)	white-box	77.47	39.64	40.06	38.4	41.7	32.3
BBUDA+MobileNet	black-box	76.64	38.25	38.74	40.6	43.2	34.8
OSUDA+MobileNet	white-box	77.32	39.37	38.92	40.1	42.0	32.5

*OSUDA (Liu et al., 2021k) with the white-box source model for UDA training is regarded as an “upper bound.”*

**Table 5:** Sensitivity analysis of the hyperparameter α.

**α**	**DSC of WholeT**
5 → 0	76.26
10 → 0	76.13
1 → 0	76.08
0	72.59
5	75.87

In addition to the HGG to LGG setting, we also proposed to adapt from the LGG to HGG setting. Similar to the HGG to LGG task, there were also domain gaps w.r.t. tumor types and the label proportion of each class. The results are shown in Table 2. Our proposed BBUDA achieved superior performance consistently.

## 5. Discussion

This work presented a UDA framework for black-box segmentation networks. The performance of the brain tumor segmentation is promising, where our framework achieved performance on par with the white-box adaptation. Therefore, our system has the potential to be applied to well-trained segmentation models in a source domain with target domain data in a range of clinical sites to combat the problem of domain shift, without the need for data sharing. Therefore, our approach enables fast, accurate, and automated lesion contouring to facilitate subsequent clinical decision processes.

Both the source domain data and network parameters are not accessible in our setting, which is a stricter requirement than typical UDA to guarantee data security, i.e., the privacy of patient data. A well-trained model usually requires large-scaled and well-labeled source domain data. However, the application of the system to different test-time institutes is likely to suffer from significant domain shifts, because of differences in study populations, imaging devices, imaging parameter settings, and subtype proportions, which lead to a significant performance drop. As a consequence, ways to make trained models available for collaborating target institutes are a key issue for the successful deployment of developed models. Among others, cross-institute data sharing can be a major difficulty in real-world applications. Recent deep inversion technologies further imposed restrictions on network parameter sharing. Therefore, our proposed framework can potentially alleviate the concerns over cross-institute medical data sharing.

The proposed knowledge distillation scheme in UDA has demonstrated its effectiveness in both cross-subtype and cross-modality tasks. The consistent loss, e.g., KL divergence, works as an efficient way to distill the knowledge in the trained black-box model. In addition, a previous study of the knowledge distillation in a single domain (Guo et al., 2020; Vu et al., 2021) also has shown that the student model learned with the distillation can be more general (Wang et al., 2021). Thus, our framework can be a viable solution to train a target domain model with a decent generalization ability.

The hyper-parameter α plays an important role in balancing between the knowledge distillation and the unsupervised learning objective. The prediction of the source domain model can provide a good initialization. The target model training with only the knowledge distillation, however, can hardly outperform the source model, i.e., teacher. Therefore, it is important to utilize the unlabeled target domain data to further improve the performance in the target domain. To this end, we linearly decreased α from 5 to 0 for all of our experiments. While changing the start value did not affect the performance significantly, the linearly decreasing scheme is an essential step to achieving our goal. We note that setting α = 0 is equivalent to only using the knowledge distillation objective. Instead, keeping α as a constant in the training cannot adjust their contribution at different training stages.

In the present work, we were able to obtain decent segmentation results in the cross-modality segmentation task, especially on EnhT. The enhanced core shown in the brain tumor MR images is due to Blood Brain Barrier disruption in high-grade glioma. A contrast agent (e.g., gadolinium) injected into the blood stream of a patient can pass to the brain parenchyma, appearing as bright regions in the post-contrast T1-weighted MR images. Recent literature shows that this information is also encoded in non-contrast MRI images (e.g., T1, T2, and FLAIR) to some extent (Ferles and Barkhof, 2021). In Preetha et al. (2021), which is one of the recent studies on T1ce synthesis, using a 3D CNN based on U-Net architecture, they reported a median Dice overlap of 28% between segmentations on synthetic and real T1ce. Although the datasets are different, they used multiple modalities as the input channels and performed training and testing in similar domains. Further investigation on synthesis and segmentation is subject to future work.

The backbones of the source and target models can be different. We only require that the input and output have a similar data structure. For example, in the cross-modality brain tumor segmentation task, our framework takes either T1-weighted or T2-weighted MRI slices as input and predicts the corresponding segmentation maps. The typical choice of the segmentation network would be FCN, PSPNet, or U-Net with ResNet or MobileNet backbones. More advanced backbones may provide better accuracy or reduce the computational cost aimed at different target applications. In addition, the backbones in some commercial black-box models may not be publicly available; our framework, therefore, enables the flexible use of a variety of backbones.

Several aspects are not fully explored in the present work. First, while we showed promising performance for brain tumor segmentation tasks with MRI, the developed framework is applied to other body parts using a variety of imaging modalities. Second, more advanced knowledge distillation and unsupervised learning methods could be analyzed beyond the current simple yet efficient framework. In addition, in the present work, we only considered a scenario, in which the source and target domains have the same segmentation classes, e.g., EnhT, ED, and CoreT in the BraTS2018 database, which is the most common case in real-world applications. Incorporating open-set UDA or out-of-distribution methods (Che et al., 2021; Liu et al., 2021a) can potentially lead to novel subtype discoveries.

## 6. Conclusion

This work proposed black-box UDA for segmentation under a realistic and meaningful scenario, presenting a practical and efficient knowledge distillation scheme with EMD pseudo labels. In particular, it provides a novel mechanism for smoothly transferring the segmentation in the source domain to the target domain with EMD to construct the pseudo label. Furthermore, unsupervised entropy minimization was incorporated into our model to improve segmentation performance. Experimental results, performed on the cross-subtype (e.g., HGG to LGG) and cross-modality (e.g., T1 to T2) adaptation tasks, demonstrated that our proposed BBUDA outperformed the source model, by a large margin, and importantly, the DSC and HD metrics of our framework were comparable to those of the white-box UDA approaches. In this work, while we only investigated brain tumor segmentation under the cross-subtype or cross-modality settings, the model could be broadly applicable to any segmentation UDA tasks using different modalities. In addition, more advanced knowledge distillation and unsupervised learning methods could be easily added to further augment performance.

## Data Availability Statement

The original contributions presented in the study are included in the article/supplementary material. Further inquiries can be directed to the first author or corresponding author.

## Ethics Statement

Ethical review and approval were not required for the study on human participants in accordance with the local legislation and institutional requirements. This research study was conducted retrospectively using human subject data made available in open access by BraTS18.

## Author Contributions

XL was involved in the conceptualization, implementation, programming, and manuscript writing. CY conceived the project and was involved in writing the manuscript. FX conceived the project and was involved in writing the manuscript. C-CK conceived and supervised the project. GEF conceived and supervised the project. J-WK conceived the project and was involved in writing the manuscript. JW conceived and supervised the project and was involved in the conceptualization and writing the manuscript. All authors read and approved the final manuscript.

## Funding

This work was partially supported by NIH R01DC018511 and P41EB022544.

## Conflict of Interest

The authors declare that the research was conducted in the absence of any commercial or financial relationships that could be construed as a potential conflict of interest.

## Publisher's Note

All claims expressed in this article are solely those of the authors and do not necessarily represent those of their affiliated organizations, or those of the publisher, the editors and the reviewers. Any product that may be evaluated in this article, or claim that may be made by its manufacturer, is not guaranteed or endorsed by the publisher.
